# Supplementary figures and images for: Genetic Diversity of the Only Natural Population of Corylus avellana L. in Kazakhstan and Prospects for Its In Vitro Conservation
Source: Biology (Basel). 2025 Oct 23;14(11):1472. doi: 10.3390/biology14111472 (PMC12650229; doi:10.3390/biology14111472)

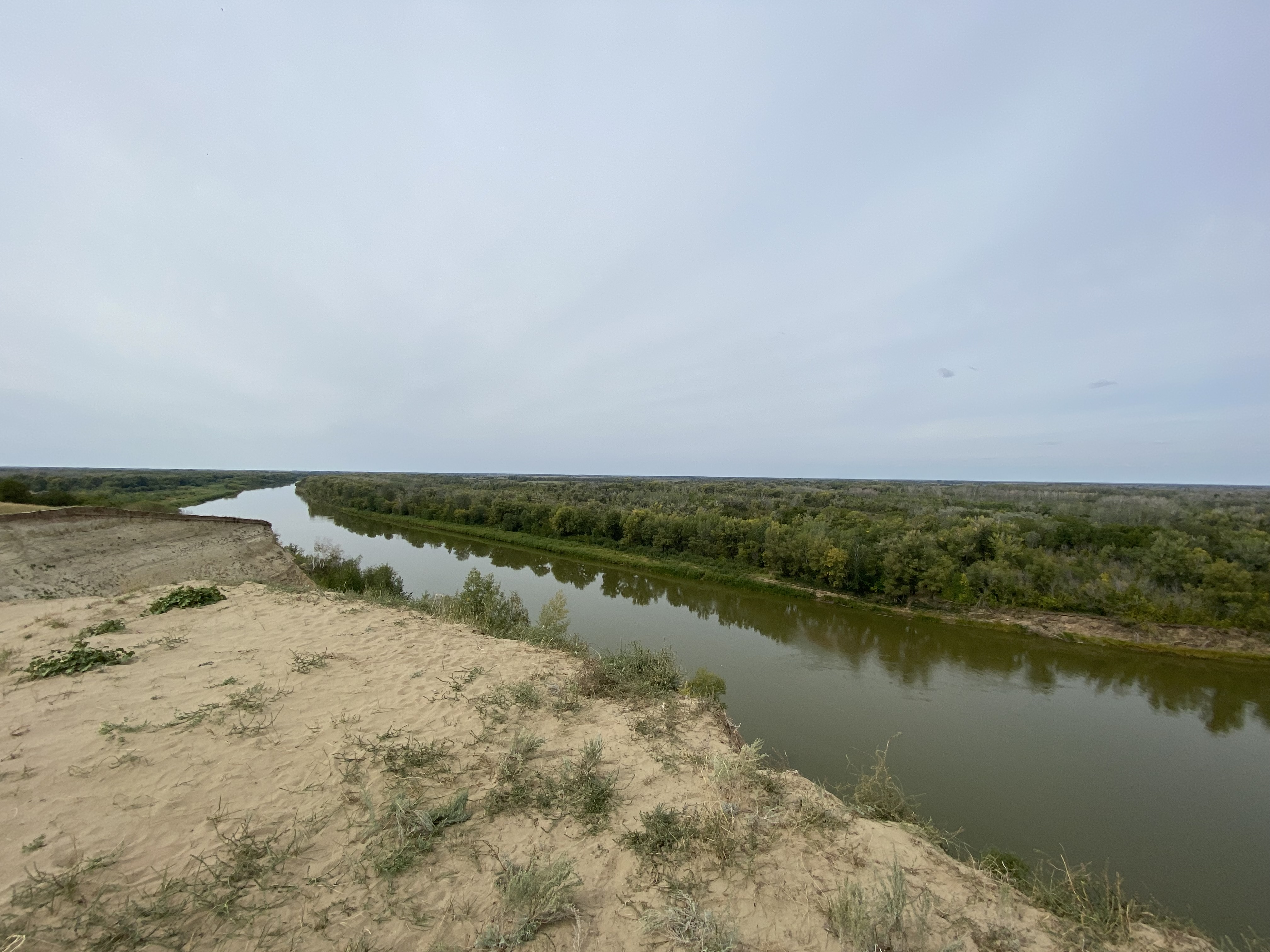

Supplement: Supplementary file 1 [file biology-14-01472-s001.zip › Figure S1.jpg]
